# Myopericarditis Associated With Marijuana Intake: A Case Report and Literature Review

**DOI:** 10.7759/cureus.39413

**Published:** 2023-05-23

**Authors:** Ali Rahman, Sura Alqaisi

**Affiliations:** 1 Internal Medicine, Northwell Health at Mather Hospital, Port Jefferson, USA; 2 Internal Medicine, Memorial Healthcare, Pembroke Pines, USA

**Keywords:** myopericarditis, smoking habits, healthy young man, marijuana abuse, marijuana

## Abstract

A 26-year-old male who endorses daily cigarette smoking and marijuana vaping presented to the emergency department with acute onset of left-sided chest pain radiating to the left shoulder. Physical examination was unremarkable, but laboratory investigations showed elevated white blood cells, cardiac biomarkers including troponin and creatine kinase, and mildly elevated C-reactive protein levels and erythrocyte sedimentation rate. Electrocardiogram displayed subtle ST-segment elevation in a diffuse pattern, leading to a diagnosis of acute myopericarditis. The patient was treated with anti-inflammatory medication and supportive care and instructed to cease cannabis use.

## Introduction

Marijuana, also known as cannabis, is a widely consumed drug across the globe. It is derived from the cannabis plant and is known for its psychoactive properties that alter the mind and behavior of individuals who consume it [1]. Marijuana is consumed in various forms, including smoking, vaporizing, edibles, and concentrates. It is estimated that over 192 million people worldwide use marijuana, making it one of the most commonly used drugs globally [1]. Marijuana contains over 100 different chemical compounds known as cannabinoids. The two most abundant cannabinoids in marijuana are delta-9-tetrahydrocannabinol (THC) and cannabidiol (CBD) [2]. THC is the primary psychoactive compound responsible for the euphoric and mind-altering effects of marijuana.

On the other hand, CBD does not have psychoactive effects and has been found to have several medicinal properties, including pain relief, anti-inflammatory, and anti-anxiety effects [2]. The physiological effects of marijuana are primarily mediated by the endocannabinoid system (ECS) [3]. The ECS is a complex network of receptors and neurotransmitters that regulate various physiological processes, including mood, appetite, pain sensation, and immune function. THC binds to the CB1 and CB2 receptors in the ECS, activating multiple signaling pathways and subsequent physiological effects [4].

Marijuana use has been shown to cause a transient increase in heart rate, typically within 10 to 30 minutes after consumption. The increase in heart rate can range from 20% to 50%, depending on the dose of THC consumed. The increase in heart rate is mainly due to the activation of the CB1 receptors in the heart, which leads to the release of norepinephrine and subsequent stimulation of the sympathetic nervous system [5]. Marijuana use has also been associated with increased blood pressure, which is thought to be due to the activation of the CB1 receptors in the blood vessels. The increase in blood pressure is typically modest, with systolic blood pressure increasing by approximately 5 to 7 mmHg and diastolic blood pressure increasing by about 3 to 4 mmHg. Marijuana use can also cause orthostatic hypotension. This effect is thought to be due to the vasodilatory effects of THC on the blood vessels [6].

The chronic cardiovascular effects of marijuana use are less understood than the acute effects. However, several studies have suggested that long-term marijuana use may be associated with an increased risk of cardiovascular disease. Marijuana use has been linked to endothelial dysfunction, where cells lining the blood vessels become less responsive to the signals that regulate blood flow. Endothelial dysfunction is thought to be an early marker of cardiovascular disease and can lead to the development of atherosclerosis [7]. Marijuana use has also been associated with an increased risk of myocardial infarction. Several studies have suggested that marijuana use can increase the risk of myocardial infarction by two to four times, particularly in individuals with preexisting cardiovascular risk factors, such as hypertension or diabetes [8]. Marijuana use has also been associated with an increased risk of stroke. One study found that marijuana use was associated with a two-fold increase in the risk of ischemic stroke [9]. The mechanism by which marijuana use may increase the risk of cardiovascular disease is not entirely clear. However, several hypotheses have been proposed. One hypothesis is that the chronic activation of the ECS by THC may lead to the development of insulin resistance, inflammation, and oxidative stress, which are all risk factors for cardiovascular disease. Another hypothesis is that marijuana use may promote the formation of blood clots, which can lead to the development of myocardial infarction or stroke. Here, we present a case of a 26-year-old man with no significant medical history who presented to the emergency department with sudden-onset chest pain found to be marijuana-induced myopericarditis.

## Case presentation

A 26-year-old male with no significant past medical history presented to the emergency department (ED) with chest pain. The chest pain had started that morning when he woke up. He described it as pressure-like, located on the left side and radiating to the left shoulder. The intensity of the pain was constant, and at its maximum, it reached 7.5-8/10. The pain was not positional or reproducible with palpation. The patient denied a recent upper respiratory infection and chest trauma or vaccination. He had no significant medical or surgical history or family history of cardiovascular diseases. However, he endorsed smoking cigarettes and vaping marijuana daily for several years, unsure about the amount he smokes. He reported smoking cigarettes and vaping marijuana as his preferred method of consumption. He denied any use of other illicit drugs or alcohol.

Upon arrival, the patient’s vital signs were within normal limits. Physical examination revealed a regular heart rhythm with no murmurs or rubs. The lungs were clear, and there was no evidence of lower extremity edema. Laboratory tests revealed an elevated white blood cell count and cardiac biomarkers, including troponin and creatine kinase (CK). The patient’s initial troponin level was 8.3ng/L, which increased to 14.6 after four hours, indicating ongoing myocardial damage. The C-reactive protein (CRP) level of 2.08 and erythrocyte sedimentation rate (ESR) of 14 were slightly elevated, indicating inflammation in the body. The D-dimer was normal in this case, ruling out the presence of a thromboembolic event (Table 1).

**Table 1:** Laboratory results. BUN: blood urea nitrogen, Pro-BNP: amino-terminal pro B-type natriuretic peptide, CK-MB: creatinine kinase, CRP: C-reactive protein, ESR: erythrocyte sedimentation rate.

Lab test	Result	Reference range
White cell count (x 10^9 ^/L)	9.9	4-11
Hemoglobin (g/dL)	14.5	12.5-16
Hematocrit (%)	41.6	41-50
Platelets (platelets per microliter)	362	140-450
Sodium (mg/dl)	140	135-150
Potassium (mg/dL)	3.3	3.5-5
Calcium (mg/dL)	8.9	8.7-10.2
Creatinine (mg/dL	0.89	0.5-1.5
BUN (mg/dL)	9	6-24
Pro-BNP (pg/mL)	187	>125
Troponin I (ng/L)	8.3 and 14.6 after 4 hours	<0.12
CK-MB (IU/L)	60	5-25
CRP (mg/dL)	2.08	>1
ESR (mm/hr)	14	0-15

**Figure 1:**
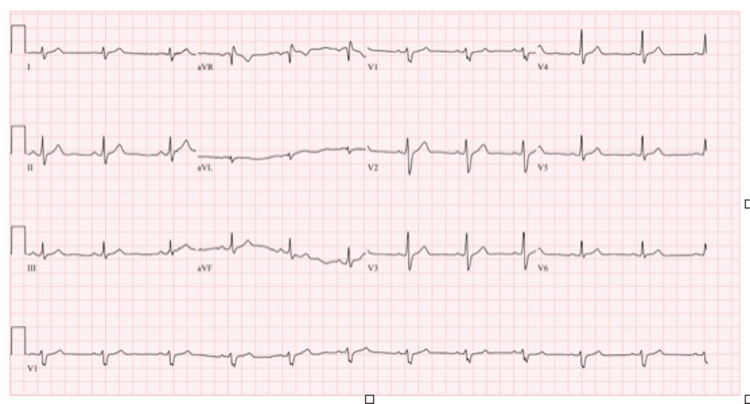
An electrocardiogram showing normal sinus rhythm, right axis deviation, and slight ST elevation on all leads.

An electrocardiogram showed normal sinus rhythm, right axis deviation, slight ST elevation on all leads, and no ischemic changes. The slight ST elevation on all leads is consistent with acute pericarditis and myocarditis. Right axis deviation could be due to right ventricular involvement or pulmonary hypertension, also commonly associated with myocarditis (Figure 1). An echocardiogram revealed evidence of minimal pericardial effusion. Based on the patient’s clinical presentation and laboratory findings, acute myopericarditis was diagnosed.

Initially, the patient was given aspirin 162mg, enoxaparin 110mg, ketorolac 30mg by mouth (PO), and potassium 30mEq PO to alleviate the chest pain and reduce the risk of blood clots. After receiving Toradol, the patient’s chest pain gradually improved and eventually resolved. The patient was then admitted to telemetry and evaluated by the cardiology team. An echocardiogram was performed, which showed an ejection fraction of 57% within normal limits. This result indicated that the patient’s heart function was normal, and there was no significant damage to the heart muscle. The patient was started on Ibuprofen 800mg three times a day for 14 days, Colchicine 0.6mg daily for three months, and Pantoprazole 40mg daily by the cardiology team to treat marijuana-induced myopericarditis. The patient was advised to follow up with cardiology regularly and to avoid using marijuana and other illicit substances to prevent future episodes of myopericarditis.

## Discussion

Pericarditis and myocarditis are inflammatory conditions affecting the heart and can present with similar symptoms. The diagnosis of these conditions is based on a combination of clinical presentation, laboratory tests, and imaging studies [10]. In pericarditis, a key clinical finding is sharp chest pain that worsens with deep breathing or changes in position. The electrocardiogram (ECG) typically shows widespread ST-segment elevation and PR-segment depression. Echocardiography may reveal pericardial effusion and thickening of the pericardium. Blood tests can show the elevation of markers of inflammation, such as C-reactive protein and erythrocyte sedimentation rate.

Similarly, myocarditis is characterized by chest pain, shortness of breath, and palpitations. The ECG may show diffuse ST-segment and T-wave changes and arrhythmias [11]. The patient’s history of daily marijuana use, his acute onset of chest pain, and elevated cardiac biomarkers suggested that his pericarditis and myocarditis were secondary to marijuana consumption.

In this case, a cardiac and/or coronary angiography may be done to further evaluate the patient's condition. However, current guidelines do not recommend the routine use of cardiac MRI or coronary angiography in the initial evaluation of acute pericarditis or myocarditis. According to the 2015 ESC Guidelines for the Diagnosis and Management of Pericardial Diseases, cardiac MRI may be considered in the diagnosis of acute pericarditis in cases of diagnostic uncertainty, such as when there is no clear evidence of pericardial effusion on echocardiography or when there is suspicion of underlying pericardial disease [12]. However, cardiac MRI is not routinely recommended in the initial evaluation of acute pericarditis, as echocardiography is usually sufficient to diagnose and monitor the condition. Coronary angiography, on the other hand, is typically reserved for cases where there is a high suspicion of underlying coronary artery disease or acute coronary syndrome. The patient described in this case had no significant past medical history, and his symptoms and diagnostic tests were consistent with acute pericarditis and myocarditis. Therefore, the likelihood of significant coronary artery disease is low, and routine coronary angiography is not recommended.

In managing myopericarditis induced by marijuana, the choice of medicines depends on the severity of the condition and the individual’s overall health. Treatment aims to reduce inflammation, manage symptoms, and prevent complications. Non-steroidal anti-inflammatory drugs (NSAIDs) such as ibuprofen are often used to reduce inflammation and relieve pain. In some cases, corticosteroids may be prescribed to reduce inflammation more effectively. Other medications, such as colchicine, may also reduce inflammation and prevent recurrences [13]. The available evidence in the medical literature on managing myopericarditis induced by marijuana is limited. Most of the data comes from case reports and small case series, which makes it difficult to draw definitive conclusions about the best treatment options. Our treatment plan aimed to reduce inflammation, relieve pain, prevent complications, and promote healing of the heart muscle. There are currently no drugs that have been specifically approved for the treatment of myopericarditis induced by marijuana. However, several potential new drugs could be beneficial. For example, recent studies have shown that cannabidiol (CBD), a non-psychoactive compound found in marijuana, has anti-inflammatory and cardioprotective effects [14]. It may be possible to develop CBD-based treatments for myopericarditis induced by marijuana that could reduce inflammation and prevent complications. Future research should focus on understanding the underlying mechanisms of myopericarditis caused by marijuana and identifying new therapeutic targets. It may also be helpful to conduct more extensive clinical trials to evaluate the efficacy and safety of different treatment options. Additionally, more research is needed to understand the long-term cardiovascular risks of marijuana use, particularly in young individuals.

## Conclusions

Marijuana use can lead to severe cardiovascular complications, including myopericarditis, even in young adults with no history of heart disease. Healthcare providers should ask about marijuana use in chest pain patients, especially young adults, as prompt diagnosis and treatment are crucial in managing myopericarditis. Early administration of anti-inflammatory medications such as ibuprofen and colchicine can help to reduce inflammation and prevent further damage to the heart muscle. Troponin levels are a useful diagnostic tool for detecting cardiac injury in patients with chest pain. The EKG is also important in diagnosing myopericarditis, as it can show slight ST elevation and right axis deviation. Collaborative care between different medical specialties is essential in managing myopericarditis. This case emphasizes the need for further studies to determine the most effective treatment options for marijuana-induced myopericarditis.
